# Spatio-temporal analysis of *Plasmodium falciparum* prevalence to understand the past and chart the future of malaria control in Kenya

**DOI:** 10.1186/s12936-018-2489-9

**Published:** 2018-09-26

**Authors:** Peter M. Macharia, Emanuele Giorgi, Abdisalan M. Noor, Ejersa Waqo, Rebecca Kiptui, Emelda A. Okiro, Robert W. Snow

**Affiliations:** 1 Population Health Unit, Kenya Medical Research Institute-Wellcome Trust Research Programme, Nairobi, Kenya; 2 Lancaster Medical School, Lancaster University, Lancaster, UK; 3 Global Malaria Programme, World Health Organization, Geneva, Switzerland; 4 National Malaria Control Programme, Ministry of Health, Nairobi, Kenya; 5 Centre for Tropical Medicine and Global Health, Nuffield Department of Clinical Medicine, University of Oxford, Oxford, UK

**Keywords:** Model-based geostatistics, Malaria, Kenya, *Plasmodium falciparum*

## Abstract

**Background:**

Spatial and temporal malaria risk maps are essential tools to monitor the impact of control, evaluate priority areas to reorient intervention approaches and investments in malaria endemic countries. Here, the analysis of 36 years data on *Plasmodium falciparum* prevalence is used to understand the past and chart a future for malaria control in Kenya by confidently highlighting areas within important policy relevant thresholds to allow either the revision of malaria strategies to those that support pre-elimination or those that require additional control efforts.

**Methods:**

*Plasmodium falciparum* parasite prevalence (*Pf*PR) surveys undertaken in Kenya between 1980 and 2015 were assembled. A spatio-temporal geostatistical model was fitted to predict annual malaria risk for children aged 2–10 years (*Pf*PR_2–10_) at 1 × 1 km spatial resolution from 1990 to 2015. Changing *Pf*PR_2–10_ was compared against plausible explanatory variables. The fitted model was used to categorize areas with varying degrees of prediction probability for two important policy thresholds *Pf*PR_2–10_ < 1% (non-exceedance probability) or ≥ 30% (exceedance probability).

**Results:**

5020 surveys at 3701 communities were assembled. Nationally, there was an 88% reduction in the mean modelled *Pf*PR_2–10_ from 21.2% (ICR: 13.8–32.1%) in 1990 to 2.6% (ICR: 1.8–3.9%) in 2015. The most significant decline began in 2003. Declining prevalence was not equal across the country and did not directly coincide with scaled vector control coverage or changing therapeutics. Over the period 2013–2015, of Kenya’s 47 counties, 23 had an average *Pf*PR_2–10_ of < 1%; four counties remained ≥ 30%. Using a metric of 80% probability, 8.5% of Kenya’s 2015 population live in areas with *Pf*PR_2–10_ ≥ 30%; while 61% live in areas where *Pf*PR_2–10_ is < 1%.

**Conclusions:**

Kenya has made substantial progress in reducing the prevalence of malaria over the last 26 years. Areas today confidently and consistently with < 1% prevalence require a revised approach to control and a possible consideration of strategies that support pre-elimination. Conversely, there remains several intractable areas where current levels and approaches to control might be inadequate. The modelling approaches presented here allow the Ministry of Health opportunities to consider data-driven model certainty in defining their future spatial targeting of resources.

**Electronic supplementary material:**

The online version of this article (10.1186/s12936-018-2489-9) contains supplementary material, which is available to authorized users.

## Background

Variations in the intensity of malaria transmission in countries requires tailoring of interventions appropriate to the corresponding level of transmission. The World Health Organization Global technical strategy for malaria 2016–2030 [1] requires National Malaria Control Programmes (NMCPs) to stratify their sub-national malaria burden based on the analysis of past and contemporary malaria data, risk factors and the environment. Cartographies of malaria risk obtained through novel and robust approaches are, therefore, required to assess the impact of control and identify areas where targeted malaria control strategies require adaptation to maximize future impact [2].

Malaria risk mapping in Kenya is not new. Maps of malaria risk were developed as early as the 1950s based on the length of the presumed malaria season [3]. In the 1970s, topography, climate, and approximations of spleen rates in children were used to classify Kenya into different endemic zones [4]. Twenty years later climate and empirical *Plasmodium falciparum* survey data were used to provide an updated cartography [5, 6]. The first attempt to apply the principles of model based geostatistics (MBG) to malaria prevalence survey data from Kenya between 1975 and 2009, at 2095 unique locations was undertaken to provide a risk map for the year 2009 [7]. This map was used to define Kenya’s unmet needs for vector control [8], future strategic planning [9] and funding [10] from 2010. This proved to be a milestone example of how applications of MBG can influence health policy planning and value for money allocation of resources to areas most in need.

However, harnessing the full value of information on malaria prevalence in time and space to provide an understanding of the fine temporal and spatial resolution changes in malaria risk at national or sub-national scales and provision of probability metrics for important programmatic policy relevant thresholds has not been attempted. Such approaches are often limited by a paucity of input data over time; Kenya however, is a country with a rich history of malaria surveys and provides a unique opportunity to explore patterns of malaria endemicity since 1990. Spatio-temporal methods were applied to understand the changing landscape of malaria transmission in Kenya since 1990 and used the statistical certainty in these models to provide insights into the future investments in control during an era of maximizing value for money.

For the first time in Kenya, a MBG framework was used to provide statistical certainty to identify areas that represent policy relevant thresholds, allowing the government to make informed choices on a more efficient future control strategy.

## Methods

### Kenya context

**Fig. 1:**
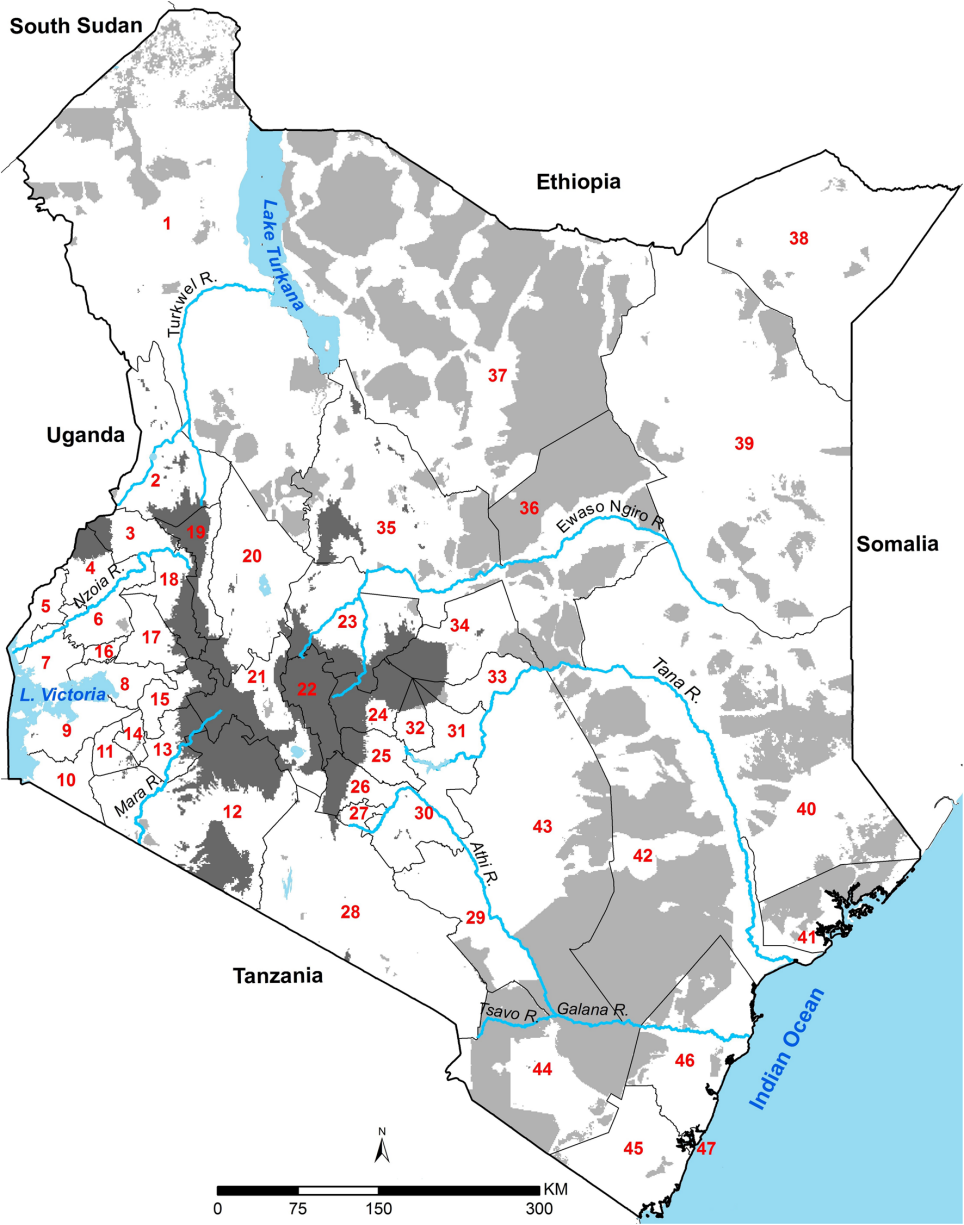
Kenya’s counties and populated malaria risk margins: 47 counties shown as dark lines with the extents of major rivers and lakes (light blue); areas unable to support *Plasmodium falciparum* transmission (dark grey) and low population density (light grey). Turkana (1), West Pokot (2), Trans Nzoia (3), Bungoma (4), Busia (5), Kakamega (6), Siaya (7), Kisumu (8), Homa Bay (9), Migori (10), Kisii (11), Narok (12), Bomet (13), Nyamira (14), Kericho (15), Vihiga (16), Nandi (17), Uasin Gishu (18), Elgeyo Marakwet (19), Baringo (20), Nakuru (21), Nyandarua (22), Laikipia (23), Nyeri (24), Murang’a (25), Kiambu (26), Nairobi (27), Kajiado (28), Makueni (29), Machakos (30), Embu (31), Kirinyaga (32), Tharaka Nithi (33), Meru (34), Samburu (35), Isiolo (36), Marsabit (37), Mandera (38), Wajir (39), Garissa (40), Lamu (41), Tana River (42), Kitui (43), Taita Taveta (44), Kwale (45), Kilifi (46), Mombasa (47). To establish the likely margins of malaria transmission, a temperature suitability index (TSI) has been used based on the monthly average land surface temperatures, the average survival of Anopheles mosquitoes and the length of sporogony that must be completed within the lifetime of one Anopheline generation, where 0 represents the inability to support transmission (dark grey) [14]. Kenya’s population is unevenly distributed within its national borders, with large areas of its land mass characterized by unpopulated areas represented by large conservation areas and deserts. Areas where population density is less than 1 person per km^2^ (light grey) [11] (Fig. 1)  were excluded from subsequent malaria risk extraction

The Republic of Kenya covers 591,971 km^2^ and lies on the equator across the great East African Rift Valley, extending from Lake Victoria to Lake Turkana and further south-east to the Indian Ocean (Fig. 1). The country has a diverse ecosystem and climate ranging from seasonal tropical coastal systems along the Indian Ocean to arid desert areas in the North and North-East, perennially hot and humid conditions around Lake Victoria and highland and mountain ranges including Mount Kenya (5199 MASL). This diversity in landscape, and the 40,487 km^2^ of national parks and conservation areas, govern the distribution of human settlement [11] (Fig. 1). In August 2010, Kenya adopted a new constitution, which decentralized policy setting and financing, including health, to 47 county governments (Fig. 1), with broad policy directions maintained at a federal level [12]. This decentralized system was formally introduced following the national election in March 2013 [13].

### Assembly o*f Plasmodium falciparum* prevalence surveys

A detailed description of the assembly of a database of malaria surveys carried between January 1980 and December 2015 in Kenya is presented elsewhere [7, 15]. These included systematic reviews of published data using free text keyword searches “*malaria*” and “*Kenya*”; searches of national ministry of health archives in Nairobi and other major centres; reviews of post-graduate theses at three major universities; school-based surveys undertaken to support the NMCP 2009–2011 [16]; national household sample surveys for nutrition or malaria in 1994, 1999, 2007, 2009/2010 and 2015; and personal communications with the extensive malaria research community in Kenya. The generosity of the local research community in sharing unpublished data makes Kenya’s malaria prevalence survey repository one of the richest in sub-Saharan Africa [15] (see “Acknowledgments”).

For each survey, details were extracted on the start and end of survey dates (month and year), age ranges (lowest and highest), sample size, numbers reported positive for *P. falciparum* infection, methods used to detect the infection and every location detail provided in the original source including the name, administrative unit, and coordinates, where available. Data were classified as points if they were individual villages, communities, schools or a collection of communities and covered an area of at most 5 km^2^. Areas covering > 5 km^2^ were classified as wide-areas. Global positioning systems (GPS) cluster coordinates collected during sample household surveys were used to re-aggregate household survey data, to increase the sampling precision by combining clusters of small sample sizes, while maintaining the 5 km^2^ criteria.

To provide a precise longitude and latitude where coordinates were not available, a variety of methods were used including reported GPS coordinates, other national digital gazetteers of populated places (cities, towns, villages), schools and health facilities [17–19]. All coordinates were checked using Google Earth (Google, 2009) to ensure that the geolocated points, were within the respective administrative boundaries of their originating source, were located on populated areas and/or settlements and not on water bodies.

### Geostatistical analysis

A geostatistical modelling framework [20–22] was used to map *P. falciparum* prevalence across Kenya between 1990 and 2015. More specifically, let *S*(*x*, *t*) denote the random effects used to account for unmeasured spatio-temporal risk factors for malaria and let *Z*(*x*, *t*) be unstructured random effects accounting for the unexplained variation within communities. Conditionally on *S*(*x*, *t*) and *Z*(*x*, *t*), the counts of positive tests for *P. falciparum* were assumed to follow mutually independent binomial distributions with number of trials *N*, corresponding to number of sampled individuals, and probability of a positive outcome *p*(*x*, *t*) at location *x* (3701) and year *t* (1990–2015) given by

$$\log \left\{ {\frac{{p\left( {x,t} \right)}}{{ 1 { - }p\left( {x,t} \right)}}} \right\} = \alpha + \beta mA + \gamma MA + S\left( {x,t} \right) + Z\left( {x,t} \right)$$

where *mA*and *MA* are the minimum and maximum age among the sampled individuals at a location x. In carrying the spatio-temporal predictions, *mA*and *MA* were set to 2 and 10 to standardize to a single age range of 2–10 years (*Pf*PR_2–10_) conventionally used for malaria risk mapping [23, 24].

The spatio-temporal random effects *S*(*x*, *t*) were modelled as a stationary and isotropic Gaussian process with spatio-temporal correlation function given by

$$cor\left\{ {S\left( {x,\;t} \right),S\left( {x^{\prime},\;t^{\prime}} \right)} \right\} = \exp \left\{ { - \left| {\left| {x - x^{\prime}} \right|} \right|/\phi } \right\}\exp \left\{ { - \left| {t - t^{\prime}} \right|/\psi } \right\}$$

where *ϕ* and *ψ* are scale parameters which regulate the rate of decay of the spatial and temporal correlation for increasing distance and time separation, respectively; ||x − x′|| is the distance in space between the locations of two communities, one at *x* and the other at *x*′; finally, |*t* − *t*′| is the time separation in years between two surveys.

The model parameters were estimated using Monte Carlo maximum likelihood implemented in the PrevMap package [25] in the R software environment (version 3.4.1). Estimates and corresponding standard errors for *Pf*PR_2–10_ were obtained from the fitted model over a 1 by 1 km regular grid covering the whole of Kenya, for every year between 1990 and 2015, exported and mapped using ArcMap 10.5 (ESRI Inc., Redlands, CA, USA). Predictions to each of the 312 months since January 1990 have not been attempted as there was insufficient monthly-gridded data to allow for such analysis.

### Model validation

The fitted spatio-temporal correlation function was validated using the following variogram-based algorithm using R software environment (version 3.4.1): (Step 1) simulate 1000 data-sets under the fitted model; (Step 2) for each simulated data-set, compute a variogram using the residuals from a non-spatial logit-linear model (i.e. by setting *S*(*x*, *t*)= *0* for all *x* and *t*); (Step 3) compute the 95% confidence interval using the resulting 1000 variograms at a predefined set of spatial distances and time separations; (Step 4) compute the variogram using the residuals from a non-spatial logit-linear model as done in step 2 but using the original data and if this falls within 95% envelope from (Step 3), then, the adopted spatio-temporal correlation was compatible with the community parasite survey data.

Cross-validation was also undertaken by holding out a 10% random sample of the survey data points selected between 1990 and 2015 to assess the predictive performance of the model. The following were computed: the correlation between observed and predicted *Pf*PR_2–10_ values, bias (mean error) representing the mean difference between the observed and predicted values, and the mean absolute error (MAE) representing the average magnitude of the errors of the absolute differences between the predictions and actual the observations [26].

### Plausibility analysis of trends

Malaria prevention and disease management milestones since 1990 in Kenya were defined by the literature, previous reviews [27–30] and major climate anomalies [31–33]. In totality, the combination of these factors might explain the changes in parasite prevalence and formed the basis of a plausibility framework [15, 34] to understand the national, annualized cycles of changing *Pf*PR_2–10_ between 1990 and 2015.

### Malaria policy relevant criteria for sub-national resource allocation and future priorities

Given the importance of county level government resource allocation for malaria, mean annual county level estimates of *Pf*PR_2–10_ were calculated by averaging the 1 × 1 km predictions among populated areas per county for the three most recent years of survey data, 2013–2015. Areas that were represented as temperature unsuitable for *P. falciparum* transmission were assigned values of 0% *Pf*PR_2–10_ [14].

Certainty of model predictions forms an important metric for NMCPs by justifying decisions on sustained, or changing control intervention policy. MBG allows for the quantification of uncertainty, which might arise from inadequate survey input data (suggesting further sampling needs) and inherent variability in small area prediction. Classifying areas into different endemic levels purely based on predicted *Pf*PR_2–10_ may lead to policy decisions that do not allow for the certainty of the *Pf*PR_2–10_ predictions [21]. Future decisions related to the choice malaria control should be based on the probability (likelihood) of an area having *Pf*PR_2–10_ below or above certain policy relevant thresholds. The choice of these thresholds should be guided by reduction targets set by the global community, malaria epidemiology, and local goals for the country of interest.

There are no formal international guidelines to countries on how thresholds of malaria risk might inform a stratified intervention response. Here, two policy relevance thresholds have been selected that might serve as valuable criteria within the Kenyan context. Areas with sustained low malaria prevalence where prevalence lies below 1% (non-exceedance probability-NEP) as an indication of pre-elimination [35], that is a transition phase which entails reorientation of malaria control programmes between sustained control and elimination stages [36]. Additionally, areas where prevalence is above 30% (exceedance probability-EP) were categorized. These mid mesoendemic areas [23] are likely to continue to yield the highest malaria burdens in the country [37] and for which intensive and sustained vector control is required.

The fitted spatio-temporal model was used to compute the probability that an area has *Pf*PR_2–10_ < 1% (NEP), and probability that an area has a *Pf*PR_2–10_ ≥ 30% (EP) across the study period and summarized for the three most recent consecutive years (2013–2015), formally expressed as

$${\text{NEP}} = {\text{Prob}}\left( {Pf{\text{PR}}_{{ 2 {-10}}} \left( {\text{x, t}} \right) < l|{\text{Data}}} \right)$$

where *l* is the prevalence threshold. A NEP close to 100% indicates that *Pf*PR_2–10_ is highly likely to be below the threshold *l*; if close to 0%, *Pf*PR_2–10_, is highly likely to be above the threshold *l*; if close to 50%, *Pf*PR_2–10_, is equally likely to be above or below the threshold *l*, hence corresponding to a high level of uncertainty. Areas likely to have a prevalence of ≥ 30% were defined by setting *l* at 30% in the preceding equation and calculating EP as

$${\text{EP}} = \left( { 1 {\text{-NEP}}} \right)$$

## Results

### Spatial–temporal mean *Pf*PR_2–10_ predictions 1990–2015

**Fig. 2:**
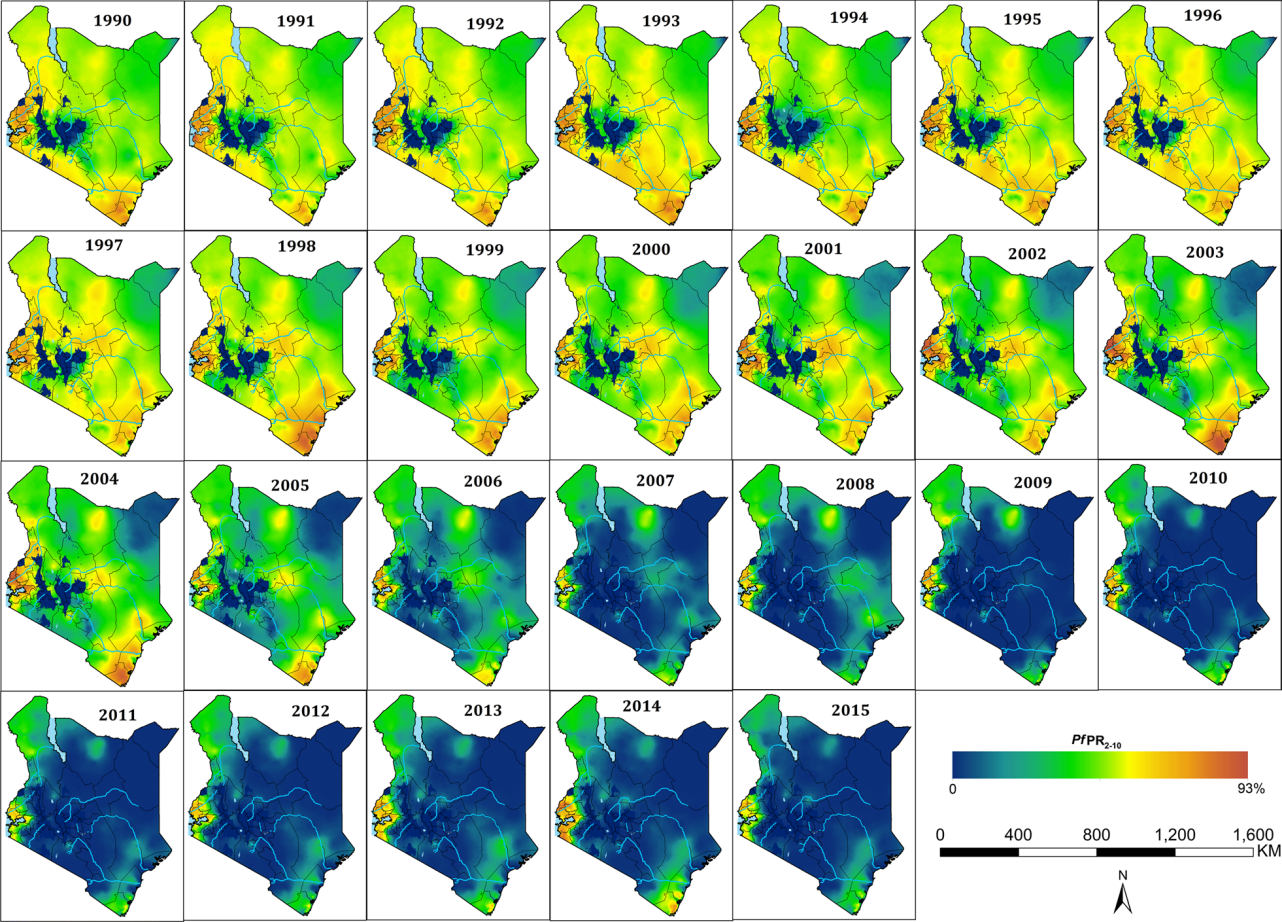
Annual predicted posterior mean community *Plasmodium falciparum* parasite rate standardized to the age group 2–10 years (*Pf*PR_2–10_) at 1 × 1 km spatial resolution from 1990 to 2015 ranging from zero (dark blue) to 93% in 2003 (dark red) in Kenya. The corresponding standard errors are provided in the Additional file 5

**Fig. 3:**
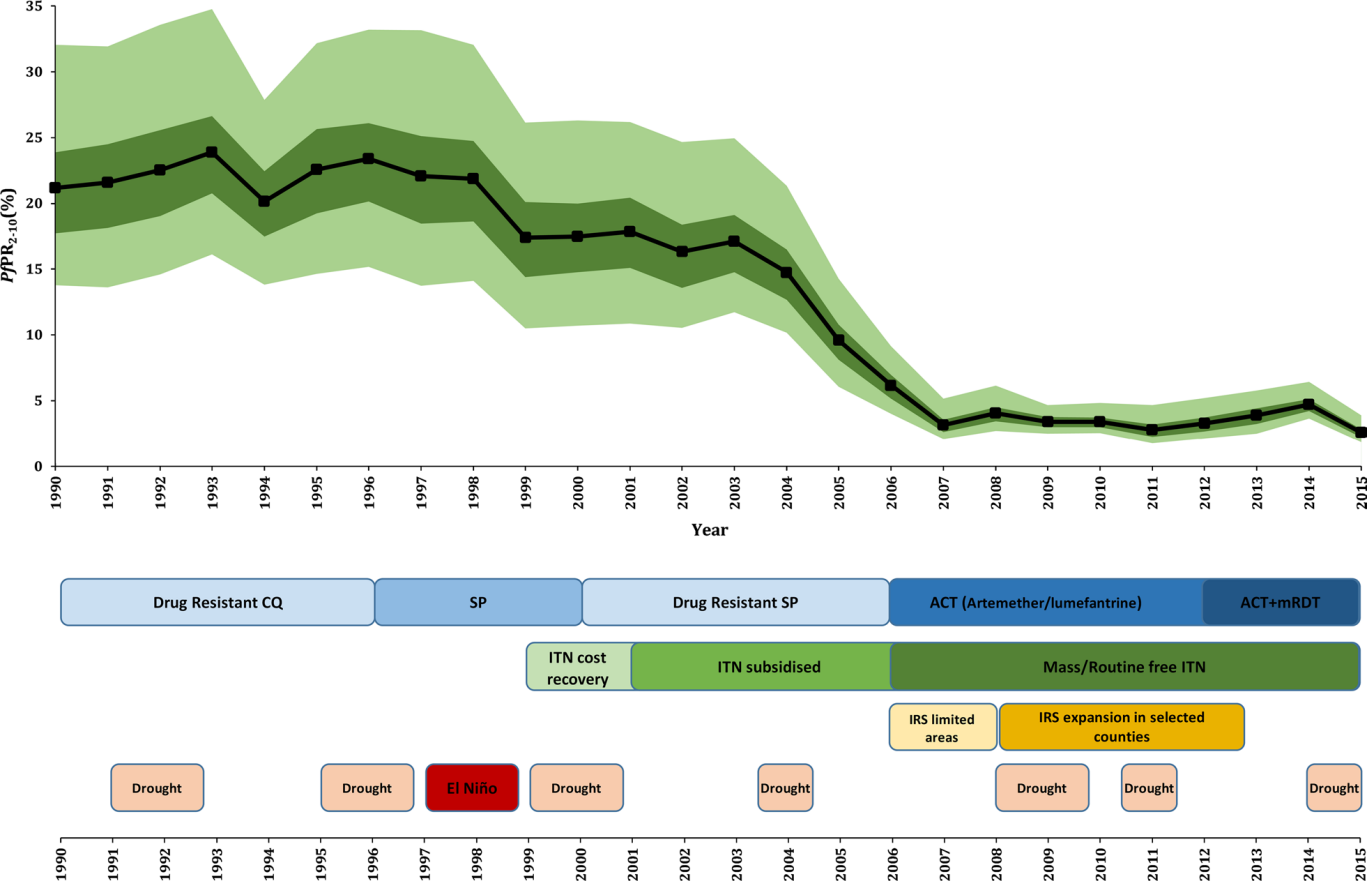
The national annual mean (black line), 2.5–97.5% (light green boundaries) interquartile credibility range (ICR) and 25–75% ICR (dark green boundaries) of the posterior *Pf*PR_2–10_ predictions in Kenya from 1990 to 2015. Unsuitable areas for malaria transmission and those with very low population were excluded in the computation of mean *Pf*PR_2–10_ and ICR. Major malaria timelines are shown in bottom panel. Blue boxes represent changing first line anti-malarial treatment and diagnostic policies using malaria rapid diagnostic tests (mRDT). Green boxes represent changing approaches to the delivery of insecticide-treated nets (ITN) through to the provision of free-of-charge of long-lasting insecticide-treated nets (LLIN) during mass campaigns in 2006, 2008, 2011/12, 2014 and 2015 alongside sustained routine delivery to infants and pregnant mothers at clinics. Indoor Residual Spraying (IRS), ( yellow boxes), has been targeted to different counties since 2006 starting in focal areas of 12 counties, by 2010/11 expanding to 16 epidemic prone and 4 endemic counties, and stopped in 2013. Peach colored boxes represent periods of drought while red represents excessive El Niño rainfall, all classified as national disasters

The final survey data was represented by 5020 surveys within 5 km^2^ at 3701 unique locations covering malaria parasite examinations of over 578,281 blood samples, between 1980 and 2015 (see Additional files 1, 2 and 3). These were used in the spatio-temporal model to generate the 1 × 1 km grids of mean posterior predictions of *Pf*PR_2–10_ 1990–2015 (Fig. 2) and summed across populated areas able to support malaria transmission for each year (Fig. 3). The results of testing the validity of the adopted spatio-temporal structure, showed that the empirical semi-variogram was within the 95% tolerance intervals (Additional file 4), thus the malaria parasite prevalence data does not show evidence against the fitted spatio-temporal geostatistical model. For each year and 1 × 1 km grid, the predicted standard errors are provided in Additional file 5. The predictive performance of the model, based on a sample of 502 validation surveys showed a high correlation between observed and predicted values of 0.86, a MAE of 7.7% and a bias of only 0.4% (Additional file 6). The model parameters are presented in Additional file 7: Table S1.

The diversity of *Pf*PR_2–10_ predictions across the country is evident from 1990 to 2015 (Fig. 2), reflecting the heterogeneity of transmission typical of Kenya, with high transmission associated with areas surrounding Lake Victoria and the Indian Ocean coastline. The highest predicted values of *Pf*PR_2–10_ were recorded in 2003 (92.5%) in Butula, Siaya county and Kinango, Kwale county; and the lowest values outside of areas unable to support transmission located in Tarbaja, Wajir county in 2011 (0.01%) (Fig. 2).

Using 1990 as a baseline, the national mean *Pf*PR_2–10_ reduced by 87.7% over a period of 26 years from 21.2% (Interquartile credibility range 2.5–97.5% (ICR): 13.8–32.1%) in 1990 to 2.6% (ICR 1.8–3.9%) in 2015 (Fig. 3). During the period 1990 and 1998, the national mean *Pf*PR_2–10_ remained largely constant (21.2%; ICR 13.8–32.1% to 21.9%; ICR 14.1–32.1%), declining slightly between 1998 and 1999, then continued at this level until 2003. The largest decline (81%) in the national mean *Pf*PR_2–10_ occurred between 2003 (17.1%; ICR 11.7–24.9%) and 2007 (3.2%; ICR 2.1–5.1%) and remained generally low thereafter. *Pf*PR_2–10_ slightly rose slowly from 2011 to 2014, following which it declined again in 2015 reaching the lowest national mean *Pf*PR_2–10_ of 2.6% (ICR 1.8–3.9%) recorded during the 26-year period of observation (Fig. 3).

The two periods of high national mean *Pf*PR_2–10_ (1990–2003) coincided with poor population coverage of vector control [27], failing chloroquine (CQ) efficacy, subsequent replacement with the long half-life, single dose sulfadoxine-pyrimethamine (SP) and its rapid increase in treatment failure rates [28, 29, 38, 39]. Interestingly, the period of greatest decline in *Pf*PR_2–10_ occurred during a period of continued use of SP, relatively poor population coverage of insecticide treated bed nets delivered on a subsidized cost-recovery basis [27] and before significant expansion of indoor residual house-spraying (IRS) in selected counties [30]. In 2006, the decision to replace SP with artemisinin based combination therapy (ACT), made in 2004, started being implemented [29], during the same year the first mass-distribution campaigns of free long-lasting insecticide-treated nets (LLIN) began and significantly increased coverage [27] and IRS began in 12 counties [30] (Fig. 3). Improved coverage of vector control and effective treatments for uncomplicated malaria continued through to 2015, however IRS was suspended in 2013, which may have resulted in the rise in *Pf*PR_2–10_ during 2014, but does not alone explain the subsequent decline in 2015 and the slight rise in *Pf*PR_2–10_ prior to IRS suspension (Fig. 3). Kenya has been characterized by periods of drought since 1990, however these have become more frequent since 2008 [32, 33] (Fig. 3). The El Niño rains which led to serious epidemics nationwide in 1997/1998 [31] occurred during periods of escalating CQ resistance and were associated with the highest period levels of *Pf*PR_2–10_ during the 1990s and early 2000s (Fig. 3).

The declining *Pf*PR_2–10_ since 2003 was not equal everywhere (Fig. 2). Areas around Lake Victoria and the southern Indian Ocean coastline, whilst shrinking in spatial extents of high *Pf*PR_2–10_ since 1990 remained high through to 2015 (Fig. 2). Conversely, areas where starting transmission intensity during the 1990s was lowest (*Pf*PR_2–10_: 9–14%), in the semi-arid North Eastern and central regions, have declined dramatically, to very low levels (< 1%) after 2006 (Fig. 2).

### Mapping areas of low and high transmission using policy relevant thresholds

**Fig. 4:**
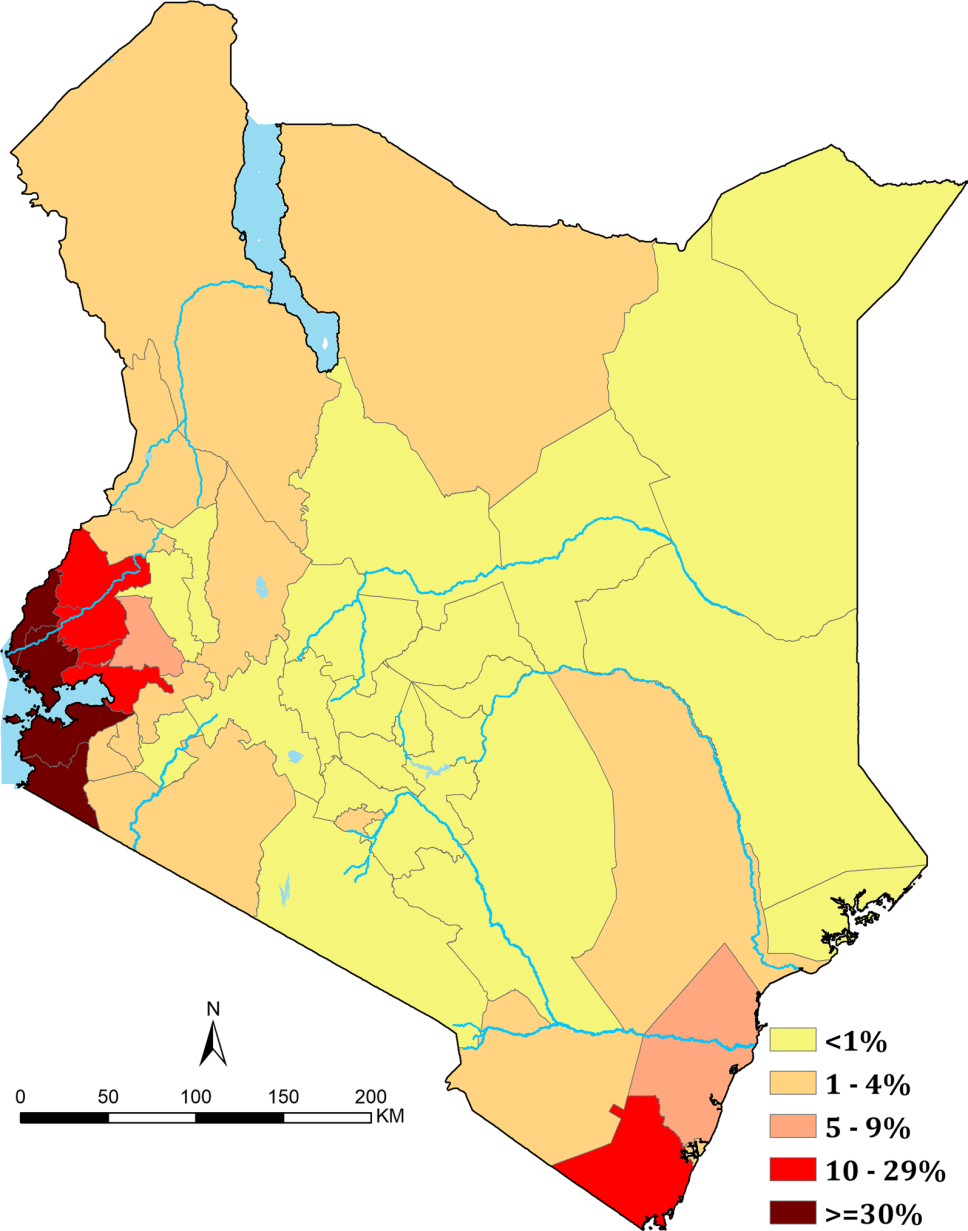
Annual county level average mean *Pf*PR_2–10_ values in populated areas 2013–2015 classified as < 1%, 1–4%, 5–9%, 10–29%, ≥ 30%

The current averaged risks of malaria in Kenya, 2013–2015 are represented by county in Fig. 4. Twenty-three (23) counties had mean predicted *Pf*PR_2–10_ of < 1% covering Central (Kiambu, Kirinyaga, Muranga, Nyandarua and Nyeri) and North Eastern (Garissa, Mandera and Wajir) regions wholly and partially in Eastern (Embu, Isiolo, Kitui, Machakos, Makueni, Meru and Tharaka Nithi), Rift Valley (Bomet, Elgeyo Marakwet, Kajiado, Laikipia, Nakuru, Samburu and Uasin Gishu) and Coastal (Lamu) region encompassing 44.3% (20.1 million) of Kenya’s 2015 population (Fig. 4).

In the 1990s, counties around the shores of Lake Victoria and the South Coast along the Indian Ocean had *Pf*PR_2–10_ values greater than 50% (hyper-holoendemic). Over the 26 years, reductions in prevalence were observed in these areas and by 2013–2015 no counties were classified as hyper-holoendemic. However, declining *Pf*PR_2–10_ was less marked over the 26 years of observation in these counties compared to countries, which started at lower transmission intensity. Four counties (Migori, Homa Bay, Siaya and Busia) had an averaged mean *Pf*PR_2–10_ of ≥ 30% between 2013 and 2015 (Fig. 4).

**Fig. 5:**
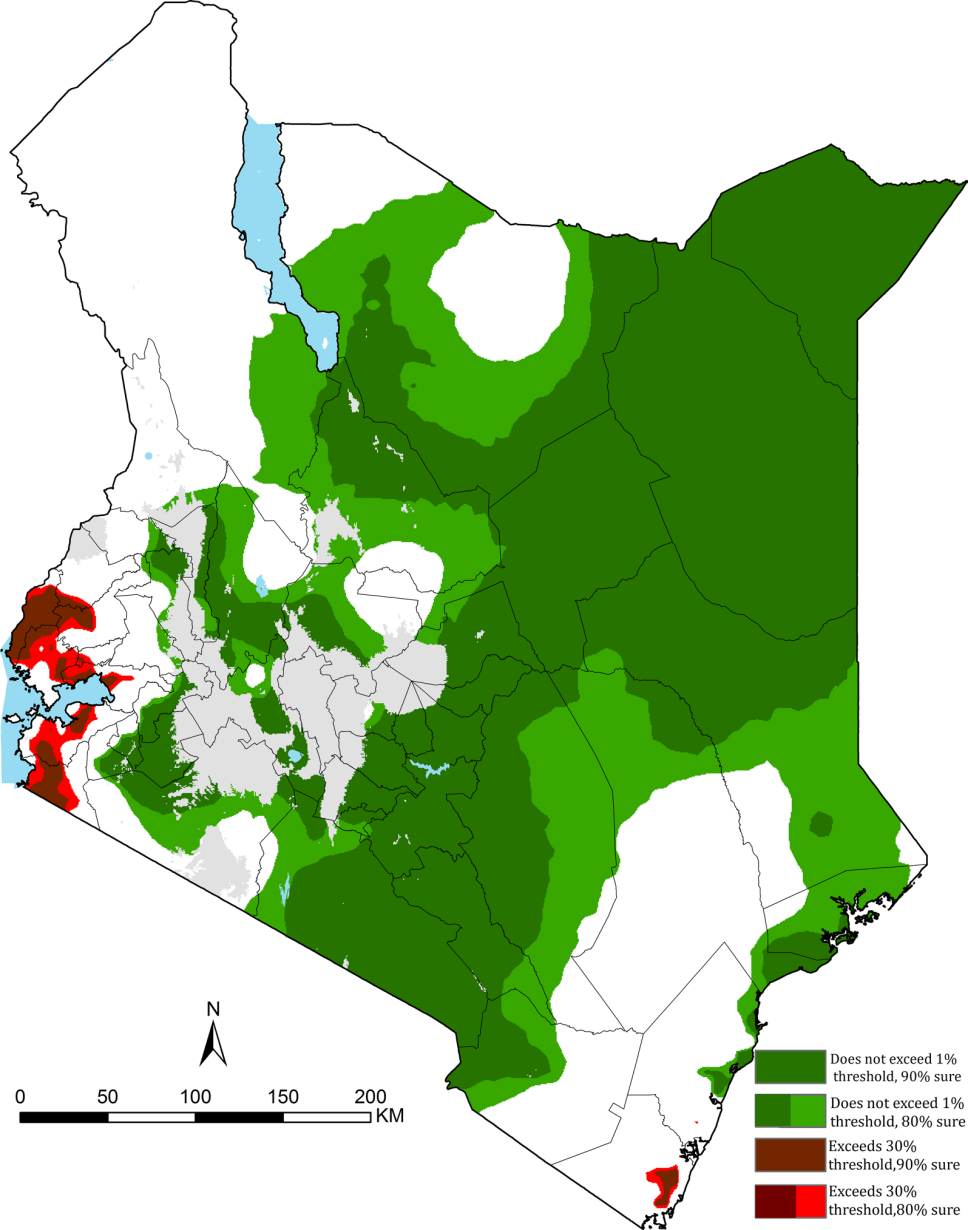
Composite of 3 years 2013, 2014 and 2015 showing areas where predicted *Pf*PR_2–10_ is less (non-exceedance probability) than 1% which were > 80% confidently predicted (light green and dark green) or > 90% confidently predicted (dark green); and areas where *Pf*PR_2–10_ is greater (exceedance probability) than 30% which were > 80% confidently predicted (light red and dark red) or > 90% confidently predicted (dark red). Areas which do not support malaria transmission are shown in grey (see Fig. 1); all other areas where transmission can occur is shown in white

The probability of the *Pf*PR_2–10_ predictions in meeting prevalence thresholds that are relevant for policy were generated for < 1% (NEPs) and ≥ 30% (EP) (Fig. 5). The maximal extents where prevalence is < 1% with ≥ 90% probability, stretches across Central, Eastern through to North-Eastern regions of Kenya between 2013 and 2015, with a slight increase in the outer margins at a less stringent probability of 80% (Fig. 5). Areas in the counties of Kilifi, Kwale, Migori, Homa Bay, Kisumu, Siaya, Kakamega, Vihiga, and Busia were likely to have a prevalence ≥ 30% at > 80% or > 90% probability levels (Fig. 5).

## Discussion

The work presented here is an extension of the 2009 map [7], incorporating more data, using a different model structure and predicting over 26 years (Fig. 2). The analysis considers a temporal presentation of how malaria transmission has changed over 26 years against the changing landscape of disease management, vector control and climate anomalies, allowing reflection on the impact of these associated covariates of *Pf*PR_2–10_ (Fig. 3). Finally, the precision in the contemporary, 2013–2015, model outputs was considered as a vital component of future decision-making (Fig. 5).

Kenya has made substantial progress in reducing infection prevalence (Figs. 2 and 3), the precise contribution of intervention versus climate are hard to disentangle. In addition, it remains difficult to distinguish whether a decrease or increase in prevalence was directly or indirectly related to an intervention being deployed or removed. Clearly, reductions were observed before the implementation of optimized treatment and vector control in 2006. The timing of this initial decline has been demonstrated at a smaller spatial scale along the Kenyan coast [40] and at a continental scale [15]. It remains uncertain as to what contributed to this initial decline in *Pf*PR_2–10_ post 2003, however reductions were accelerated and sustained after 2006, which shows continued reductions in national infection rates (Fig. 3), and continued shrinking of the high-intensity areas (Fig. 2). This occurred during a period when sustained efforts to ensure continued replacement of LLINs as part of mass campaigns and routine delivery to pregnant women and infants were high, and treatment regimens for uncomplicated malaria switched to ACT (Fig. 3). The slight rise in 2014 cannot be entirely explained by the stopping of IRS in 20 counties in 2013, since the rise had already started in 2011. This was also observed on the Kenyan coast [40] where IRS has not been implemented and nationally returned to levels similar to those during IRS campaigns in 2015.

The heterogeneous nature of *P. falciparum* transmission in Kenya continues to be reflected in present-day (2013–2015) descriptions of risk nationwide. A large swathe of the country is occupied by areas predicted to have a *Pf*PR_2–10_ less than 1% with a probability of at least 80%, covering approximately 68% (297,497 km^2^) of the populated areas and 61% (27.8 million people) of Kenya’s 2015 population. At a higher probability (≥ 90%) at least half (51%) of Kenya’s populated areas, occupied by 53% of Kenya’s population has a prevalence of less than 1%. In such populations where the infection prevalence over the period 2013–2015 is < 1%, should be an indication for possible migration to a pre-elimination phase by the NMCP [35]. In these areas the coverage of good quality laboratory and clinical services, reporting and, surveillance should be reinforced. Strengthening of surveillance systems will allow quick detection of infections and prompt treatment with effective anti-malarials to prevent onward transmission within this band of low transmission [36].

The unexpected *Pf*PR_2–10_ observed in Nairobi (1.1%), might be due to a combination of locally acquired and imported malaria [41]. A population-based infectious disease surveillance over a 5-year period (2007–2011) in Nairobi (Kibera slums) reported that about two-thirds of patients with malaria had traveled to high malarious areas of Western Kenya [42]. It seems reasonable to assume that Nairobi continues to be exceptionally low prevalence, and where transmission occurs likely limited to the peripheral areas, for example, at a probability of 90%, 68% of county was likely to have a prevalence < 1% while at a probability of 80% the entire county was likely to have < 1% *Pf*PR_2–10_ 2013–2015 (Fig. 5).

There continues to be areas of Kenya, which over the last 26 years appear to be intractable to current coverage levels, and approaches to vector control. Areas that on average continue to support *Pf*PR_2–10_ levels of transmission ≥ 30% are located around Lake Victoria, inland toward the highlands and along the southern coast of the Indian Ocean (Fig. 2). While smaller in their geographic extent (8515 km^2^), compared to low transmission, these areas encompass 3.9 million people, 8.5% of Kenya’s 2015 population. The counties affected by this elevated level of *Pf*PR_2–10_ transmission are Kilifi, Kwale, Migori, Homa Bay, Kisumu, Siaya, Kakamega, Vihiga, and Busia (Figs. 2 and 4), however, none of the counties are entirely covered by the 80% exceedance probability that it completely belongs to this endemicity class (Fig. 5). It would, therefore, seem reasonable to expand vector control since the current coverages are still low and below NMCP targets, and introduce other possible innovative approaches to parasite control in these nine counties and might include the use intermittent preventive treatment of infants [43] and/or the use of RTS, S vaccine [44].

Spatio-temporal geostatistical models of sparse malaria input data have used multiple, dynamic [45] or long-term averaged covariates [46] in the prediction of malaria risk. However, caution is urged in the use of multiple covariates in malaria risk mapping. The inclusion of covariates (climate, land use, social economic status and intervention) to assist predictions at locations without data presume: clearly defined and uniform biological relationship with prevalence; the veracity of the averaged or temporally varying covariate data is often not tested; and including covariates related to intervention coverage precludes any further analysis of the impact of intervention on infection prevalence. The present Kenya analysis avoids the use of covariates because, unlike many other countries, there is a large volume of empirical input data, and the empirical prevalence data are a product of all the possible covariate influences of climate and intervention coverage, allowing a plausibility analysis of the role of climate and intervention, thus avoiding circularity. Caution should be extended beyond Kenya, countries without empirical data on prevalence should not be modelled on the basis of presumed covariate associations with malaria or prediction made in data rich countries to years beyond the last available empirical data.

The novelty of non-exceedance probabilities will allow the NMCP in Kenya, and other malaria endemic countries, to implement control measures that are congruent to malaria risk. This may involve re-orientation of resources allowing optimal utilization of funds in a time of competing health agendas and limited resources. The global momentum is to stratify national malaria control because a blanket cover of intervention is no longer appropriate in increasingly heterogenous settings [1]. The work presented here highlights the statistical value of NEPs and EPs as a tool for future policy formation.

## Conclusion

Kenya has made substantial progress in reducing *P. falciparum* infection prevalence over time. The declines in transmission intensity were heterogeneous in nature over the 26 years. However, the reductions were witnessed before the implementation of optimized treatment and vector control. Areas confidently classified to have prevalence < 1% calls for a possible migration to control strategies suited for a pre-elimination phase. Conversely, in the areas which over the last 26 years seem to be intractable to current levels of vector control coverage will require expansion of vector control and use of other innovative approaches to control both the parasite and vector.

## Additional files

**Additional file 1.** Summary of malaria parasite surveys data used in the analysis.

**Additional file 2.** The temporal and frequency distribution of communities surveyed for malaria infection between 1980 and 2015 in Kenya.

**Additional file 3.** The locations of communities surveyed for malaria infection in Kenya between 1980 and 2015.

**Additional file 4.** Spatio-temporal structure validation.

**Additional file 5.** Spatio-temporal variation of the standard errors.

**Additional file 6.** Model predictive performance assessment through cross validation.

**Additional file 7.** The Monte Carlo maximum likelihood parameters from the fitted spatio-temporal geostatistical model.

## Abbreviations

EP: exceedance probability
MAE: mean absolute error
MASL: mean height above sea level
NMCP: National Malaria Control Programme
NEP: non-exceedance probability
*Pf*PR_2–10_: *Plasmodium falciparum* parasite rate standardized to the age group 2–10 years
