# Supplementary material for: Spatio-temporal analysis of Plasmodium falciparum prevalence to understand the past and chart the future of malaria control in Kenya
Source: Malar J. 2018 Sep 26;17:340. doi: 10.1186/s12936-018-2489-9 (PMC6158896; doi:10.1186/s12936-018-2489-9)
Supplement: Supplementary file 1 — Additional file 1. Summary of malaria parasite surveys data used in the analysis. [file 12936_2018_2489_MOESM1_ESM.docx]

**Additional File 1**

**Data summary**

Among all survey data located between 1980 and 2015, spatial coordinates of four survey data points (0.08%), three in 1992 and one in 1994 could not able identified. 87 surveys classified as wide areas were excluded. The 5020 geo-coded surveys were sourced from peer-reviewed journals (17.5%), conference abstracts (7.1%), postgraduate theses (2.3%), malaria indicator surveys of 2007, 2010 and 2015 (13.3%), Ministry of Health reports of schools and nutritional surveys (24.6%) and other archived non-governmental or research reports (35.2%). Over 76.7% of the survey data were further disaggregated or provided as unpublished reports.

The locations of the surveyed communities were located using GPS recordings (76.7%), national digital place name gazetteers (28%), Google earth (2.7%), Encarta (1.6%) and personal communication (0.1%). 3687 (73.5%) of the surveys had a sample size of 50 or more, 2285 (45.5%) a population of 100 or more while only 296 (5.9%) had a sample size of fewer than 20 individuals. The median sample size across all 5020 surveys was 90 (IQR: 47,110). 1691 (33.7%) of the surveys used Rapid Diagnostics Kits (RDTs): Paracheck (Device and dipstick) (1190); CareStart (263); Rapid Uni-Gold (103); OptiMal (91); ICT (26) and SD Bioline (18) with 636 (37.6%) of the total RDTs confirmed by slide. 66.3% (3329) of the surveys employed microscopy with one confirmed by Polymerase Chain Reaction (PCR). The maximum and minimum recorded *Pf*PR_2-10_ were 99.8% and 0.0 % respectively while the median was 11.8% (IQR: 0.0,36.1%).
