# Supplementary material for: Spatio-temporal analysis of Plasmodium falciparum prevalence to understand the past and chart the future of malaria control in Kenya
Source: Malar J. 2018 Sep 26;17:340. doi: 10.1186/s12936-018-2489-9 (PMC6158896; doi:10.1186/s12936-018-2489-9)

**Additional File 2**

**Figure S1:** The frequency and temporal distribution of 5020 communities surveyed for malaria infection between 1980 and 2015 at 3701 unique locations selected for analysis in Kenya


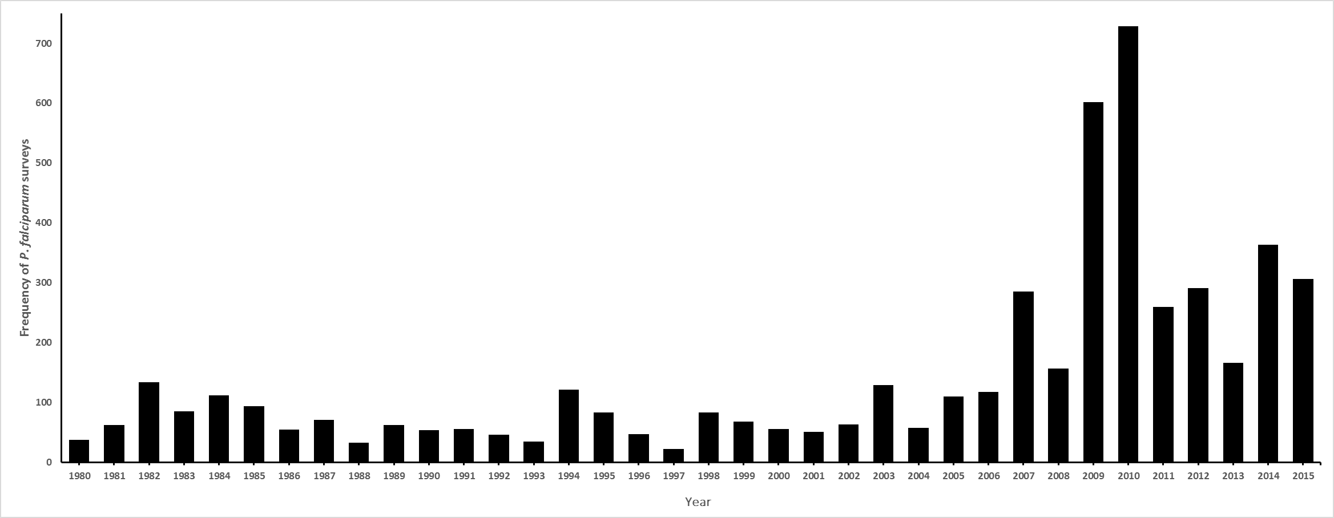

Supplement: Supplementary file 2 — Additional file 2. The temporal and frequency distribution of communities surveyed for malaria infection between 1980 and 2015 in Kenya. [file 12936_2018_2489_MOESM2_ESM.docx]
