# Supplementary material for: Spatio-temporal analysis of Plasmodium falciparum prevalence to understand the past and chart the future of malaria control in Kenya
Source: Malar J. 2018 Sep 26;17:340. doi: 10.1186/s12936-018-2489-9 (PMC6158896; doi:10.1186/s12936-018-2489-9)

**Additional File 3**

**Figure S2:** The locations of 5020 communities surveyed for malaria infection at 3701 unique locations in Kenya between 1980 and 2015. **A)** Highest *Pf*PR_2-10_ values on top; **B)** lowest *Pf*PR_2-10_ values on top.


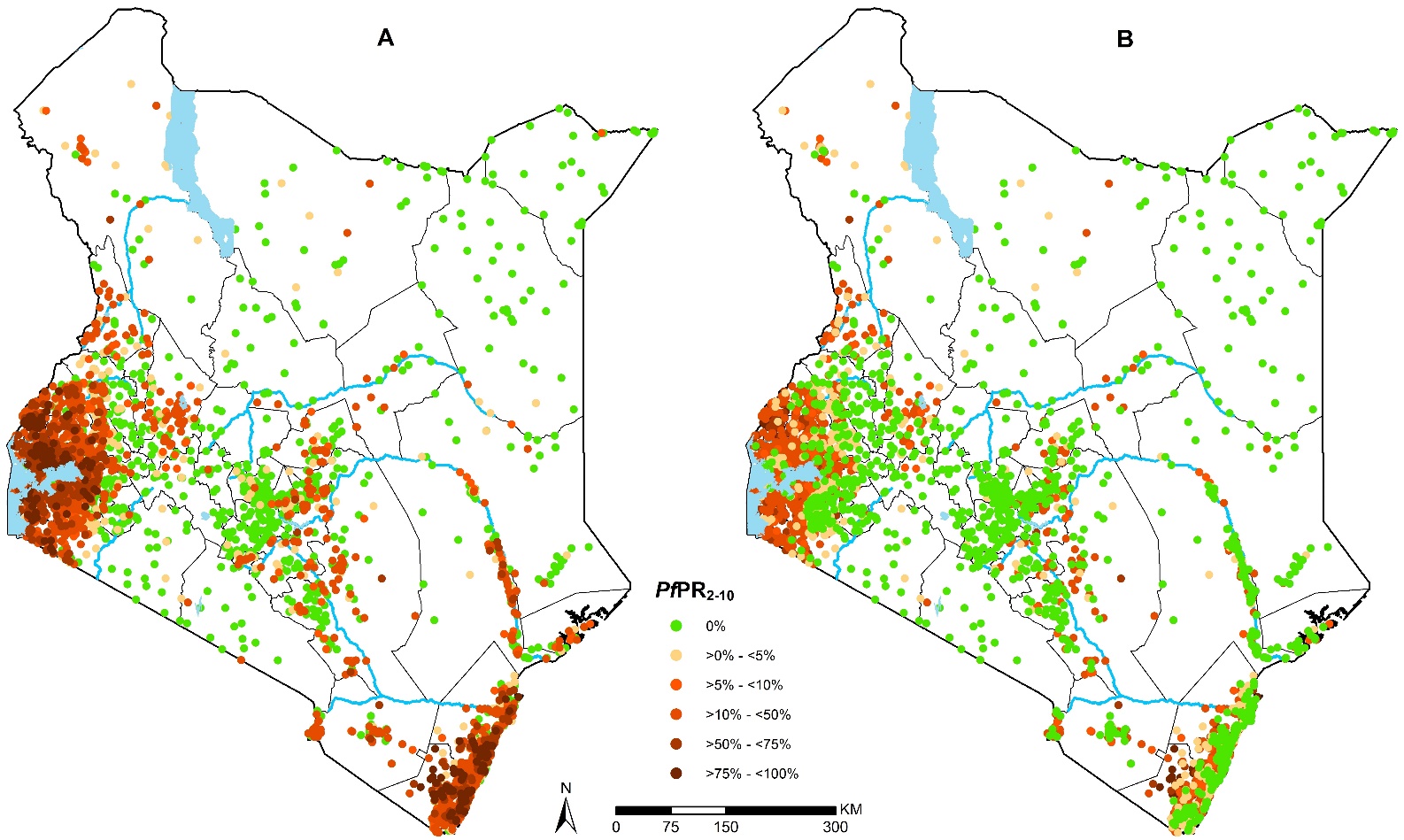

Supplement: Supplementary file 3 — Additional file 3. The locations of communities surveyed for malaria infection in Kenya between 1980 and 2015. [file 12936_2018_2489_MOESM3_ESM.docx]
