# Supplementary material for: Spatio-temporal analysis of Plasmodium falciparum prevalence to understand the past and chart the future of malaria control in Kenya
Source: Malar J. 2018 Sep 26;17:340. doi: 10.1186/s12936-018-2489-9 (PMC6158896; doi:10.1186/s12936-018-2489-9)

**Additional File 4**

**Spatio-temporal structure validation**

The validity of the adopted spatio-temporal structure used in the modelling exercise was tested using steps outlined in the main text. This is significant especially when identifying areas where prevalence lies below (Non-Exceedance Probability-NEP) or above (Exceedance probability-EP) pre-defined thresholds. The results of this exercise are shown in Figure 1 below. Since the empirical semi-variogram (solid line) falls within the 95% tolerance intervals (dashed lines), then the adopted covariance model was compatible with the malaria parasite prevalence data implying that the results of NEPs and EP are valid.

**Figure S3**: Model validation plot, the solid line is the variogram based on the residuals from a non-spatial model (empirical semi variogram). The dashed lines are the 95% confidence intervals generated under the fitted spatio-temporal geostatistical model.


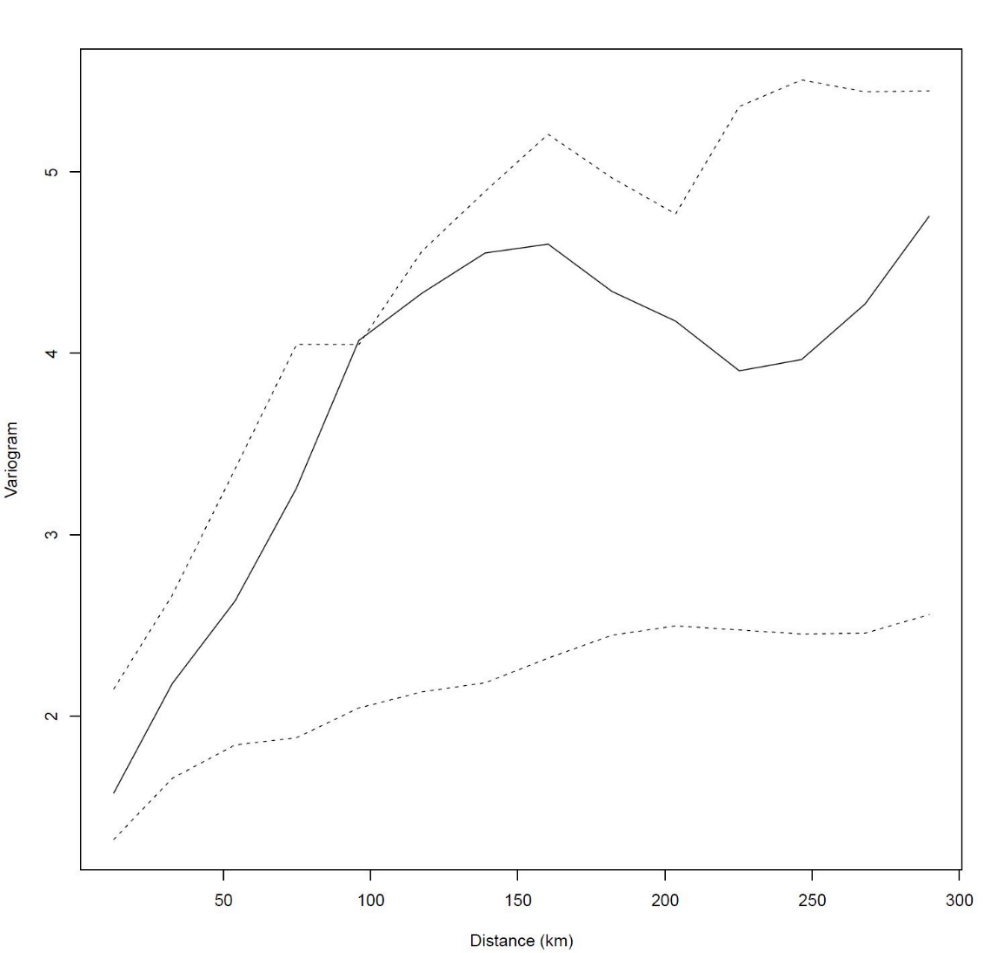

Supplement: Supplementary file 4 — Additional file 4. Spatio-temporal structure validation. [file 12936_2018_2489_MOESM4_ESM.docx]
