# Supplementary material for: Spatio-temporal analysis of Plasmodium falciparum prevalence to understand the past and chart the future of malaria control in Kenya
Source: Malar J. 2018 Sep 26;17:340. doi: 10.1186/s12936-018-2489-9 (PMC6158896; doi:10.1186/s12936-018-2489-9)

**Additional File 5**

The precision of the predictive mean was characterized using standard errors (SE). Figure 1 shows the spatio-temporal variation of SE at 1 by 1 km resolution between 1990 and 2015. Its variation mirrors that of the mean *Pf*PR_2-10_ and the amount of data available by year. Large SE are associated with large values of *Pf*PR_2-10_ and smaller sample size (1990-2004). The SE falls as the sample size increases (2005-2015), which implies that the variation observed in *Pf*PR_2-10_ is not due to chance.

**Figure S4:** Standard errors associated with each pixel between 1990 and 2015 for the posterior mean *Pf*PR_2-10_ in Kenya


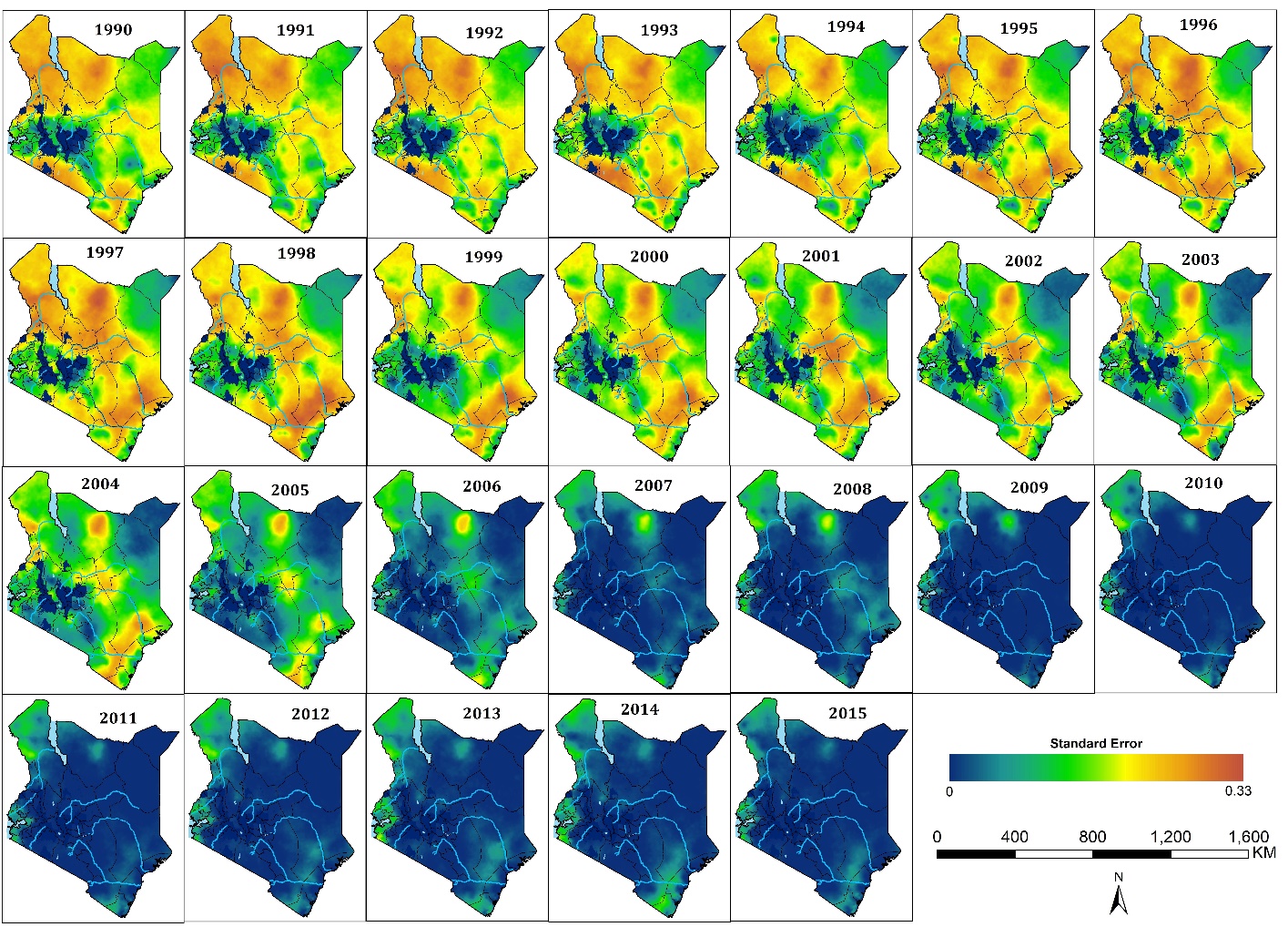

Supplement: Supplementary file 5 — Additional file 5. Spatio-temporal variation of the standard errors. [file 12936_2018_2489_MOESM5_ESM.docx]
