# Supplementary material for: Spatio-temporal analysis of Plasmodium falciparum prevalence to understand the past and chart the future of malaria control in Kenya
Source: Malar J. 2018 Sep 26;17:340. doi: 10.1186/s12936-018-2489-9 (PMC6158896; doi:10.1186/s12936-018-2489-9)

**Additional File 6**

The linear association between the observed and the predicted *Pf*PR_2–10_ was assessed for a 10% subset of the data points (n =502) which were randomly selected. As shown in Figure 1 below, the linear association was strong with a correlation of 0.86.

**Figure S5:** Validation plot showing a scatter of the association between the observed *Pf*PR_2–10_ versus the predicted *Pf*PR_2–10_ for a 10% subset of the data points (n =502) randomly selected.


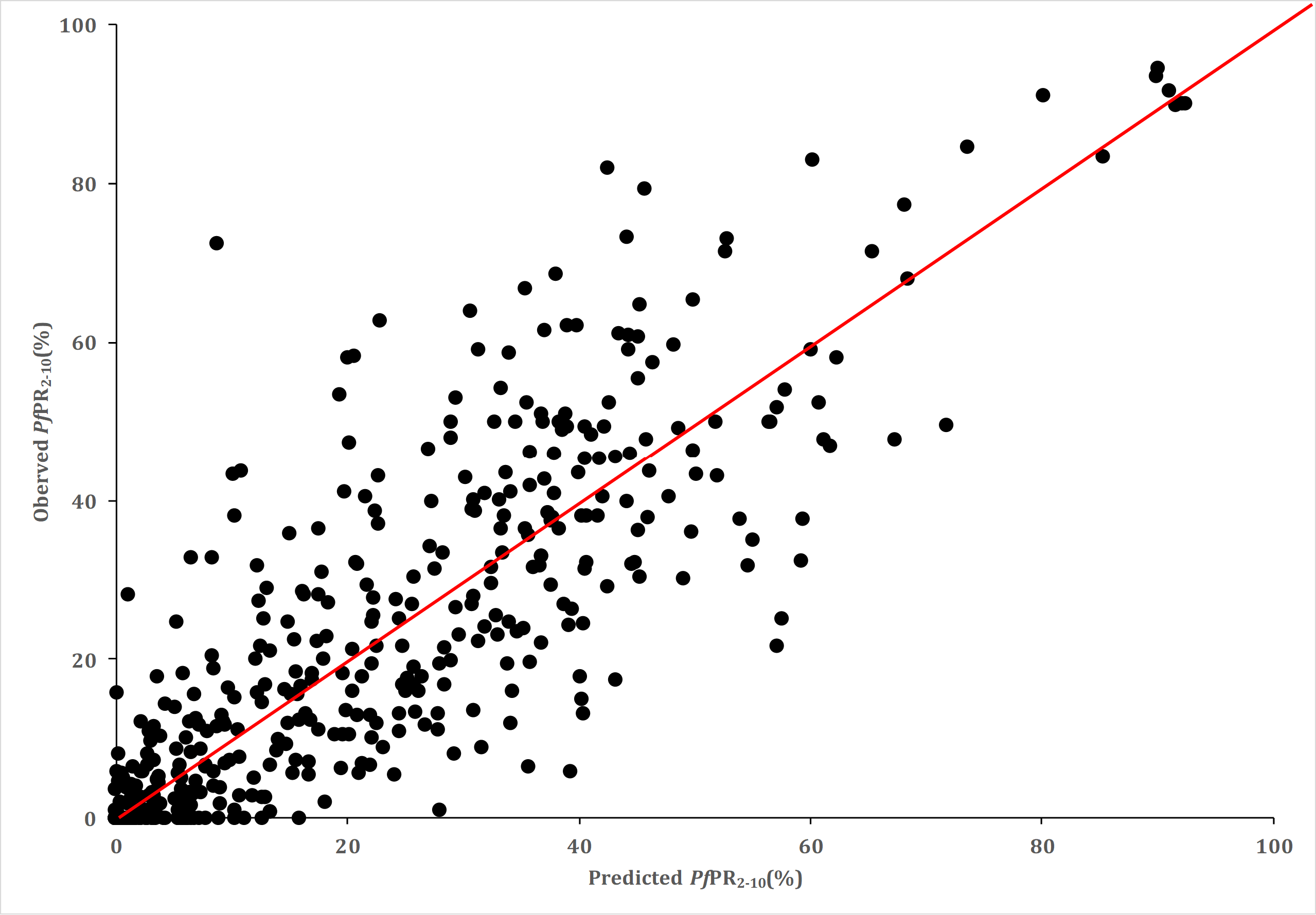

Supplement: Supplementary file 6 — Additional file 6. Model predictive performance assessment through cross validation. [file 12936_2018_2489_MOESM6_ESM.docx]
