# Supplementary material for: Spatio-temporal analysis of Plasmodium falciparum prevalence to understand the past and chart the future of malaria control in Kenya
Source: Malar J. 2018 Sep 26;17:340. doi: 10.1186/s12936-018-2489-9 (PMC6158896; doi:10.1186/s12936-018-2489-9)
Supplement: Supplementary file 7 — Additional file 7. The Monte Carlo maximum likelihood parameters from the fitted spatio-temporal geostatistical model. [file 12936_2018_2489_MOESM7_ESM.docx]

**Additional File 7**

**Table S1**: The Monte Carlo maximum likelihood parameters and their corresponding 95% confidence intervals from the fitted spatio-temporal geostatistical model.

| Parameter | **Estimate** | **95% CI** |
| --- | --- | --- |
| Intercept | -3.733 | -5.031 to -2.435 |
| Lower Age | -0.065 | -0.085 to -0.045 |
| Upper Age | -0.003 | -0.004 to -0.001 |
| Sigma Squared ($\sigma^{2}$**)** | 7.868 | 5.703 to 10.854 |
| Phi () | 150.971 | 113.412 to 200.969 |
| Tau squared ($\tau^{2}$) | 0.403 | 0.285 to 0.569 |
| Psi ($\psi$) | 12.539 | 9.582 to 16.408 |
